# Core Shell Investigation of 2-nitroimidazole

**DOI:** 10.3389/fchem.2019.00151

**Published:** 2019-04-02

**Authors:** Paola Bolognesi, Vincenzo Carravetta, Luca Sementa, Giovanni Barcaro, Susanna Monti, Preeti Manjari Mishra, Antonella Cartoni, Mattea C. Castrovilli, Jacopo Chiarinelli, Sanja Tosic, Bratislav P. Marinkovic, Robert Richter, Lorenzo Avaldi

**Affiliations:** ^1^CNR-Istituto di Struttura della Materia, Area della Ricerca di Roma 1, Montelibretti, Italy; ^2^CNR-Istituto per i Processi Chimico Fisici, Pisa, Italy; ^3^CNR-Istituto di Chimica dei Composti Organo Metallici, Pisa, Italy; ^4^Stored and Cooled Ions Division, Max Planck Institute for Nuclear Physics, Heidelberg, Germany; ^5^Dipartimento di Chimica, Sapienza Università di Roma, Rome, Italy; ^6^Dipartimento di Scienze, Università degli Studi di Roma 3, Rome, Italy; ^7^Institute of Physics, University of Belgrade, Belgrade, Serbia; ^8^Elettra Sincrotrone Trieste, Area Science Park, Trieste, Italy

**Keywords:** XPS, NEXAFS, mass spectrometry, 2-nitroimidazole, DFT, MCFCS calculations

## Abstract

Tunability and selectivity of synchrotron radiation have been used to study the excitation and ionization of 2-nitroimidazole at the C, N, and O K-edges. The combination of a set of different measurements (X-ray photoelectron spectroscopy, near-edge photoabsorption spectroscopy, Resonant Auger electron spectroscopy, and mass spectrometry) and computational modeling have successfully disclosed local effects due to the chemical environment on both excitation/ionization and fragmentation of the molecule.

## 1. Introduction

Inner shell electrons are localized on specific molecular sites, due to their “atomic-like” nature, and, because they are affected by the chemical environment, can provide details on specific molecular bonds. By means of the tunable and monochromatic X-ray synchrotron radiation, it is possible to deposit well-defined quanta of energy in selected molecular sites and to probe specific electronic configurations through core shell excitation and ionization. The question that arises then, is whether the molecular fragmentation induced by inner shell processes is also site-selective, i.e., affected by the “localization” of the core hole, as well as, in the case of photoabsorption, by the localization and character of the excited orbital. For more than three decades (Eberhardt et al., 1983; Hanson, 1990), this question has been addressed by using different experimental techniques, which combined ion, electron, and electron-ion coincidence spectroscopies to study both core ionization (Rühl et al., 1993; Nagaoka et al., 2011) and excitation (Rühl et al., 1993; Okada et al., 2003; Tanaka et al., 2006; Céolin et al., 2008; Bernini et al., 2012; Lin et al., 2014, 2015; Salén et al., 2014). The features in the absorption spectra are used to “localize” the electron vacancy, through a suitable choice of the photon energy, without detecting the photoelectron as in the case of core ionization. It has been argued that a localized core excitation could be used as a sort of “molecular knife”(Tanaka et al., 2001) to induce controlled bond breakings near the atom of the primary excitation. It is usually observed that the molecular fragmentation following core excitation is strongly influenced by both the molecular site of the initial excitation and the character of the excited molecular orbital. In fact, significant variations in the fragment branching ratios have been observed across different excitation thresholds. In some cases, (Ueda et al., 1999; Liu et al., 2005), an ultrafast molecular fragmentation takes place on a time scale comparable to the electronic decay time of the core hole. In such cases, the process being driven by the elongation of specific bonds adjacent to the core excited atom will be dominated by the formation of specific fragments “cut” around the atomic site of excitation. However, in most cases the “molecular knife” interpretation of the site-selective bond scission is questionable, due to the existence of many fast electronic relaxation channels ending with the removal of one or more electrons from the valence shell and subsequent delocalization of the excess energy. Therefore, the “degree of localization” of the primary excitation is rapidly lost. Indeed, some authors suggested that the site-specific fragmentation patterns could be explained as a “memory effect”(Larkins, 1990; Habenicht et al., 1991), where the electronic relaxation is driven by the overlap between the core-hole and the valence orbitals in the final state and depends on the electron density of the valence orbitals near the excitation site. This was confirmed by electron-ion coincidence experiments on 2Br-pyrimidine (Bolognesi et al., 2015), where the preferential formation of certain fragments observed at selected resonant core excitation energies was found to depend on the overlap between inner shell excited and the valence ion states, while a direct correlation between a site-selected excitation and a bond breakage could not be established. Within this scenario, site-selective fragmentation patterns are mainly due to selective electronic relaxation mechanisms, which lead to the population of dissociative single and doubly ionized valence states, that are fragmented following their own peculiar pattern. The goal of the present study is to explore the electronic structure of 2-nitroimidazole (2NIM) in the core excitation and ionization regions at the C, N, and O K edges by experiments and quantum mechanics calculations and to investigate the possible correlations between these core excited electronic states and the following molecular fragmentation.

The nitroimidazole molecule (*C*_3_*H*_3_*N*_3_*O*_2_) is made of an imidazole ring, (*C*_3_*H*_4_*N*_2_), where a hydrogen atom is replaced by the nitrogen dioxide (*NO*_2_) group bound to a carbon atom. For the X-ray photoelectron spectra (XPS) a qualitative approach, based on the known properties of the building blocks of nitroimidazoles, provides an excellent guideline for the interpretation of the spectrum, confirmed by the differential Density Functional Theory (ΔDFT) calculations. The 2NIM core levels can be related to those of the two building blocks by a predictable chemical shift due to the higher electronegativity of the nitro group. The near-edge X-ray absorption fine spectra (NEXAFS) accurately assigned by quantum mechanics calculations, provide a description of the electronic structure of the main core excited states. Finally, the mass spectrometry experiments allow to follow the fate of the dissociative core excited/ionised states by evolution of the partial ion yields across the different thresholds.

The photoionization and photofragmentation properties of nitroimidazole isomers, including 2NIM, have already been investigated in the valence region for the neutral (Bolognesi et al., 2016; Cartoni et al., 2018) and protonated (Feketeová et al., 2015b) molecule. The 4(5)NIM isomers have also been studied by XPS and NEXAFS (Feketeová et al., 2015a) as well as by ion-ion coincidence experiments (PIPICO) (Itälä et al., 2017). Sections 2 and 3 describe the experimental and theoretical methods, while section 4 is devoted to the description and discussion of the XPS, NEXAFS, and mass spectrometry measurements. Section 5 contains summary and conclusions.

## 2. Experimental

The experiments were performed at the Gas Phase photoemission beamline (Blyth et al., 1999) of the Elettra synchrotron (Trieste, Italy) using the end station, already described in previous studies, for photoemission and mass spectrometry measurements (Bolognesi et al., 2015). Briefly, the radiation source of the Gas Phase beamline is an undulator (Diviacco et al., 1992) that provides fully linearly polarized radiation in the energy range from 13.5 to about 900 eV. Monochromatization of the radiation is provided by five interchangeable gratings and the energy selected photon beam reaches the interaction region in a spot size of a diameter of about 300 μ*m*. The experimental apparatus is a high vacuum chamber hosting a hemispherical analyzer (VG 220i) and a custom made Wiley McLaren (Wiley and McLaren, 1955) time-of-flight (TOF) spectrometer mounted opposite to each other at the magic angle with respect to the polarization axis of the photon beam. The hemispherical analyzer, which mounts six channeltron detectors for parallel acquisition, was used to measure the XPS spectra of the C, N, and O (1s) orbitals of 2NIM at about 90 eV above their respective ionization thresholds, with an overall energy resolution of 0.3 eV. Each XPS spectrum required an acquisition time of about 10 h, with a typical counting rate of the order of 10 counts/s. The chosen photon energies are sufficiently far from the ionization thresholds so that post-collision interaction effects can be neglected. The TOF spectrometer was operated with an extraction field of 700 Vcm^−1^ and antisymmetric polarization of the repeller/extractor electrodes (Directed Energy Inc. model PVM4210). The operation mode can be either a continuous extraction, for the measurement of the total ion yield in the NEXAFS spectra, or a pulsed extraction for mass spectrometry, using a pulse generator (Stanford Research DG535) at 1 kHz frequency. In the NEXAFS spectra the photon energy resolution was 20 and 50 meV at the C, N, and O K-edges, respectively. The measured yields were normalized to the photon beam intensity variation read by a photodiode placed at the end of the beamline. The mass spectra were measured at several photon energies in the C, N, and O K near-edge regions, with a step size of 0.25 eV and a typical acquisition time of 800 s per point. In the data analysis, the background was subtracted to each raw mass spectrum and then the intensity of each m/z fragment was evaluated as the sum of yields within the time-of-flight range of interest (corresponding to about m/z ± 0.5). Then, the branching ratio of each fragment was obtained by normalization to the total ion yield.

Both the XPS and NEXAFS spectra were calibrated according to the well-known references of CO_2_ (Wight and Brion, 1974; Tronc et al., 1979, 1980; Hatamoto et al., 2007) (C(1s)^−1^ at 297.6 eV, O(1s)^−1^ at 541.3 eV, C(1s) → π^*^ at 290.77 eV and O(1s) → π^*^ at 535.4 eV) and N_2_ (Thomas and Shaw Jr, 1974; Sodhi and Brion, 1984) (N(1s)^−1^ at 409.9 eV and N(1s) → π^*^ at 400.87 eV), inserted as diffuse gases in the vacuum chamber. The 2NIM sample (molar mass 113 Da and 98% purity) was purchased from Sigma Aldrich. The powder was introduced into the vacuum chamber in a crucible and heated to about 80°C to be sublimated for gas phase analysis. The background pressure was 2 x 10^−8^ mbar and a cold finger was used to reduce contamination from background residual water.

## 3. Theory

Calculations were carried out for both ionization (XPS) and excitation (NEXAFS) of the core shells of the three C, three N, and two O atoms of 2NIM. The photoemission spectra were modeled by all-electron differential methods fully including electronic relaxation (ΔHF) and partially including electronic correlation (ΔDFT) by using the DALTON code (Angeli et al., 2013). With the same code, we carried out accurate multiconfigurational self-consistent field (MCSCF) calculations to assign a peculiar feature appearing in the oxygen K-edge XPS spectrum due to a shake-up process. The Restricted Active Shell (RAS) approximation, describing the electron correlation in the highest 12 valence levels, and the medium size Ahlrichs-VTZ basis set (Schäfer et al., 1992) were adopted. A valence electron method introduced recently (Iannuzzi and Hutter, 2007), with the use of a potential to describe the relaxation of the core hole, was employed for the simulation of the NEXAFS spectra. These calculations were performed with cp2k (Lippert et al., 1999) within the all-electron implementation of the Gaussian Augmented Plane Wave (GAPW) (Krack and Parrinello, 2000) method. The Kohn-Sham (KS) wave-functions were projected on a set of Gaussian contracted functions. We employed two different atomic basis sets: aug-cc-pVTZ (Dunning Jr, 1989) for the molecular structure optimization and aug-cc-pVQZ to improve the accuracy of the higher excited states of NEXAFS spectra.

The computational efficiency of the GAPW method relies on an auxiliary electronic density that is partitioned in an electronic part, smoothly varying between atoms and in an electronic part, rapidly varying close to the nuclei. For a fast evaluation of the Coulomb and Exchange potentials, a plane-wave expansion is adopted to project the former, whereas combinations of localized atomic functions describe the latter. Thus, beyond Gaussian basis sets, we used plane waves with an energy cut-off of 300Ry to expand the smooth part of the auxiliary electronic density. The BLYP (Becke, 1988; Lee et al., 1988) density functional was chosen for the exchange and correlation part of the KS hamiltonian. We calculated the NEXAFS spectra by following the protocol described by Iannuzzi and Hutter (2007) which is based on the direct calculation of both the excitation energy and the dipole transition element between the selected core orbitals and a certain number of virtual orbitals obtained through a full-core-hole transition potential (Jayawardane et al., 2001; Hetényi et al., 2004) on the adsorbing atoms. This method predicts accurate relative positions of the main NEXAFS features. In order to get the absolute energy scale for the full set of excitations at a given K-edge, we performed DFT calculations to estimate the energy difference between the ground state and the first core-excited state. The Stieltjes Imaging method (Langhoff, 1980; Cacelli et al., 1991) was applied to the discretized excitation spectrum, followed by a convolution with a Gaussian (FWMH = 0.2 eV) to mimic both the finite lifetime of the excited states and the limited experimental resolution.

## 4. Results and Discussion

The presentation of the results is organized in three subsections, devoted to the XPS, NEXAFS, and mass spectrometric experiments, respectively. For the XPS and NEXAFS experiments, a qualitative approach, based on the spectroscopy of imidazole and nitrogen dioxide molecules, guided a first assignment of the different features of the spectra. This was then fully validated by the ΔHF, ΔDFT, ΔMCSCF, and TDDFT calculations: the discussion of the observed chemical shifts unravels the role played by the nitro group in the stabilization of the imidazole ring atoms. The theoretical prediction of the NEXAFS spectra allows to disentangle the contribution of the different non-equivalent atoms in each absorption spectrum and to analyze the charge distribution in the lowest unoccupied molecular orbitals, LUMO, and LUMO+1. The last subsection reports the results of the time of flight mass spectra measured at several photon energies in the C, N, and O near K-edge regions and the discussion of the site-selective molecular fragmentation.

### 4.1. The XPS Spectra

**Figure 1:**
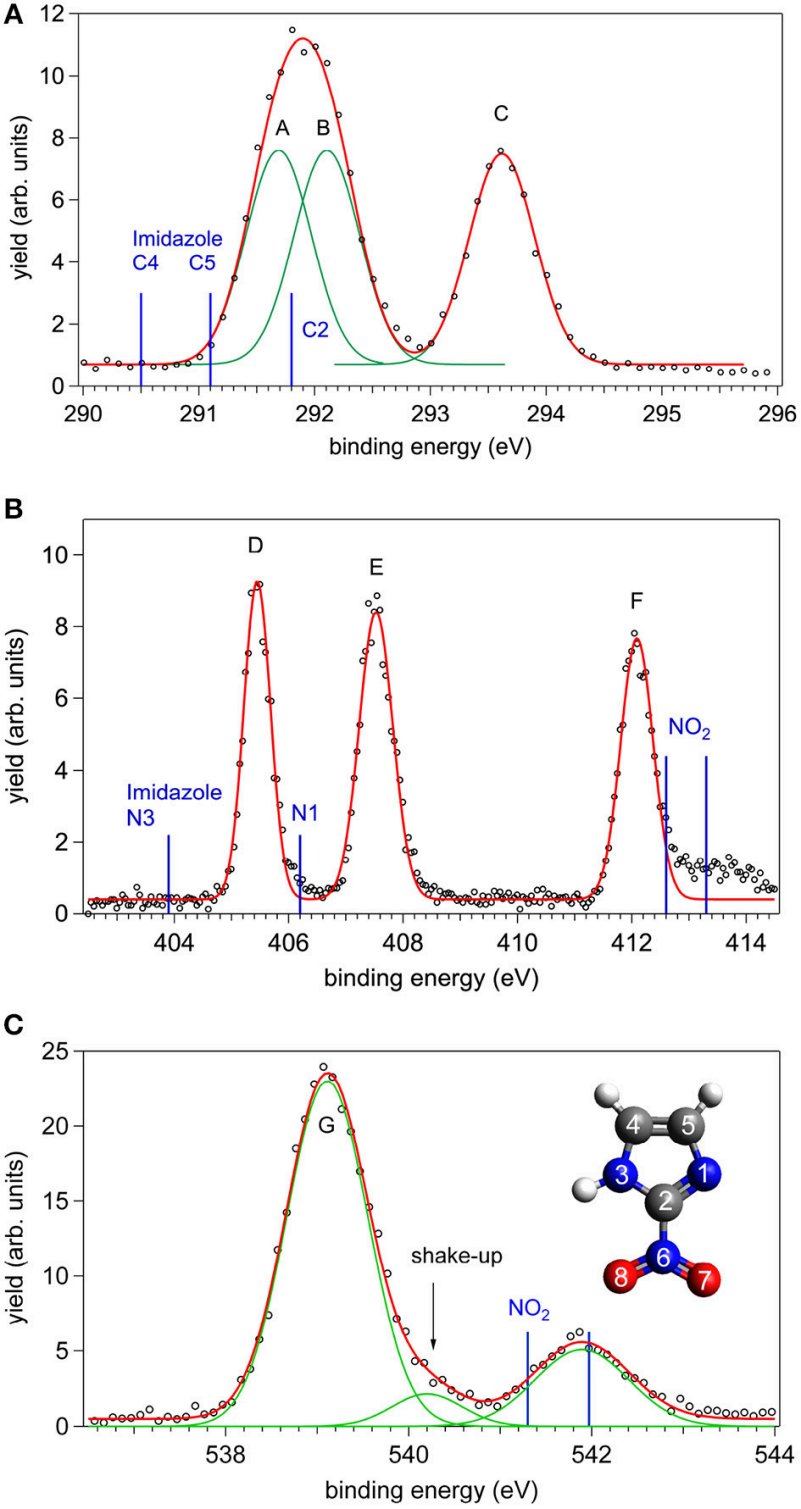
The C, N, and O XPS spectra of 2NIM measured at a photon energy about 90 eV above their respective ionization thresholds: experimental points (black circles) and best fitting with Gaussian functions (red and green lines). The energy position of the XPS lines of imidazole and nitrogen dioxide are also shown as blue bars in the C, N, and O cases, respectively (see also Figure 2 and references in the caption for source data). In the bottom panel, the inset shows the structure of the 2NIM molecule and its atoms numbering.

The XPS spectra of 2NIM at the C, N, and O K edges are shown in Figure 1. The main C and N spectral features were tentatively fitted with three Gaussian functions with the same area, corresponding to the number of non-equivalent atoms of the same species. The results are indicated as peaks A to C for carbon, and D to F for nitrogen. In the O case, the two non-equivalent atoms O7 and O8 are expected to be non-degenerate. However, the calculated splitting of about 100 meV (see Table 1) combined with the vibrational broadening makes the two peaks unresolvable in the XPS measurement within the present experimental resolution. Therefore, only the average position of the two contributions is reported in the experimental data and the O (1s) peak is labeled G.

**Table 1:** Comparison of present experimental and theoretical core ionization potentials of 2NIM with equivalent experimental and theoretical data for the 4- and 5NIM isomers (Feketeová et al., 2015a), imidazole (Becke, 1988), and nitric dioxide (Jayawardane et al., 2001).

**Site**	**2NIM**	**2NIM**	**4(5)NIM**	**4NIM**	**5NIM**	**Imidazole**	**${\text{NO}}_{2}^{*}$**
	**Expt(eV)**	**Th (eV)**	**Expt (eV)**	**Th (eV)**	**Th (eV)**		
C4	A, 291.69(2)	292.59	292.6	292.41	291.83	290.5	–
C5	B, 292.11(2)	293.14	293.1	292.16	292.89	291.1	–
C2	C, 293.62(2)	294.97	293.4	292.71	292.77	291.8	–
N3	D, 405.45(2)	406.37	406.37	405.40	405.31	403.9	–
N1	E, 407.53(2)	408.27	408.27	407.67	407.67	406.2	–
N6	F, 412.09(2)	413.66	413.66	411.28	411.73	–	412.6(5)
							413.3(5)
O7	G, 539.10(2)	538.63	538.5	538.09	538.50	–	541.3(5)
O8		538.74		538.19	538.47	–	542.0(5)

The latter two molecules can be considered as building blocks of the nitroimidazole family of molecules (see text). Numbers in parenthesis represent the uncertainty of the present fitting procedure. *NO_2_ being an open shell molecule, the core ionization leads to two (${}^{3}A_{1}$ and ${}^{1}A_{1}$) states due to spin coupling.

**Figure 2:**
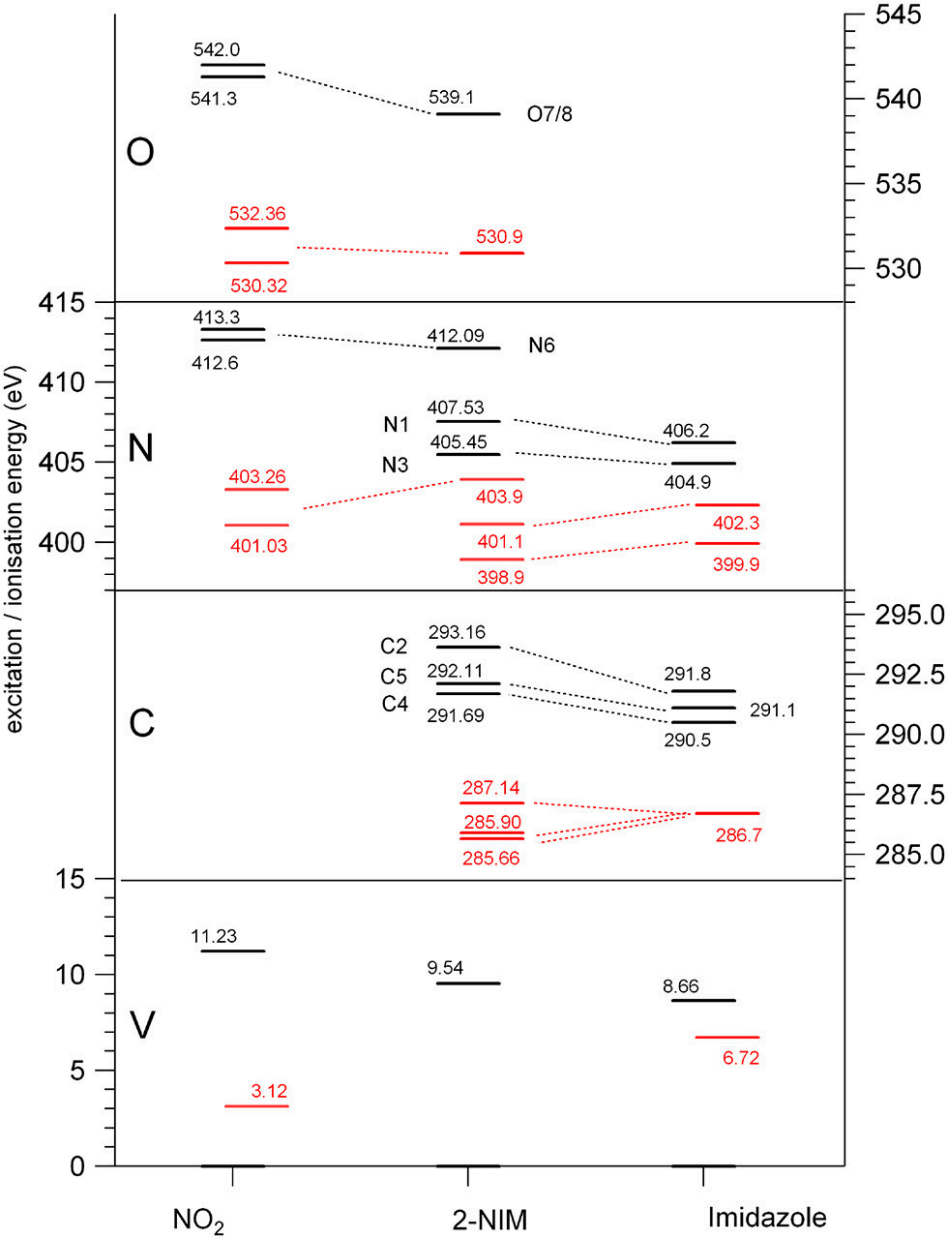
Diagram of the binding energies (black) and of the lowest (LUMO) excitation energies (red) at the O, N, and C K edges and at the valence shell (Kimura, 1981; Schwell et al., 2008; Cartoni et al., 2018), for nitrogen dioxide (Davis et al., 1973; Au and Brion, 1997; Jayawardane et al., 2001) (left), 2NIM (center) and imidazole (right) (Apen et al., 1993; Thomason et al., 2015) molecules.

A first qualitative assignment of these XPS spectra is suggested by the energy positions of the main XPS bands for imidazole (Apen et al., 1993; Thomason et al., 2015) and nitrogen dioxide (Davis et al., 1973; Jolly et al., 1984), i.e., the building blocks of the nitroimidazole molecule. The core binding energies (BE) of these building blocks are represented by the vertical bars in Figure 1, and the corresponding values are reported in Table 1, together with the binding energies of 4- and 5-NIM isomers from Feketeová et al. (2015a). An overview of the 2NIM, imidazole and nitrogen dioxide XPS binding energies is also displayed in the diagram of Figure 2. Based on the qualitative analogies among the nitroimidazole isomers and their building block molecules, we assign the 2NIM XPS peaks A to G for increasing binding energies to the C4, C5, C2, N3, N1, N6, O7/8 atoms, respectively. This qualitative assignment is fully validated by our ΔDFT calculations, also reported in Table 1. Apart from an average shift of about 1.1 eV for the C and N spectra and –0.42 eV for O, there is good agreement between the theoretical predictions and both the experimental observations, and the qualitative assignment provided by the “building block approach.”

The three C(1s) XPS peaks in all nitroimidazoles show a clear influence of the presence and specific location of the NO_2_ group on the imidazole ring. In fact, all C(1s) binding energies (black levels in Figure 2) are chemically shifted toward higher values with respect to those of the corresponding atoms in the imidazole molecule. These positive shifts are due to the electron withdrawing of the NO_2_ group, producing a charge transfer from the imidazole ring to the nitro group, which reduces the electronic relaxation around a core hole on the ring atoms, increasing their binding energies. In 2NIM, this effect is more pronounced on the C2 atom, which is directly involved in the C2-NO_2_ bonding. The experimental data provided 1.82 eV for the C2 chemical shift, to be compared with an average shift of 1.1 eV for C4 and C5 with respect to imidazole. It should be noted that according to the Koopmans theorem (KT), ground state HF calculations predict chemical shifts of 2.5, 1.3, and 1.6 eV for the C2, C4, and C5 atoms, respectively, going from imidazole to 2NIM. ΔSCF calculations, fully including electron relaxation around a specific core hole, predict instead 2.2, 1.7, and 0.7 eV respectively, to be compared to experimental values of 1.8, 1.2, and 1.0 eV (see Figure 1). It is clear, from this comparison, that an interpretation of the chemical shift as due to an “initial state effect” (KT) is only a rough approximation and can be erroneous in the prediction of the relative size of the chemical shift for non-equivalent atoms in the molecule. The inclusion of electronic relaxation around the core hole (final state effect) can instead provide a reliable estimation of the chemical shift.

For the N(1s) XPS spectrum, the comparison between 2NIM and its building blocks is more complex; in fact, it is necessary to distinguish the case of the ring atoms N1 and N3 of the imidazole and the N6 atom of the NO_2_ molecule. The building block values are significantly separated in energy (around 7 eV), clearly manifesting the influence of the chemical environment on the core binding energy, with the nitrogen atoms surrounded by the two strongly electronegative oxygen atoms in NO_2_, or by carbon atoms, when embedded in the imidazole ring. In 2NIM, the N3 and N1(1s) electron binding energies, similar to the C case, present a positive shift with respect to imidazole, while N6 presents a negative shift with respect to nitrogen dioxide, i.e., shifting toward smaller binding energies. This can be explained by considering the same effect already discussed in the C case where, in the C2-NO_2_ bond, the nitro group withdraws electron charge mostly from the C2 atom, but also, by inductive effect, from the entire imidazole ring. Therefore, the reduced screening on the N3 and N1 ring atoms increases their (1s) electron binding energies while on the N6 atom of the nitro group this has the opposite effect, increasing the shielding and therefore decreasing the N6(1s) binding energy with respect to the NO_2_ isolated molecule.

In the binding energy of the O(1s) electrons, similarly to the N6 case, there is a significant negative shift of about –2.5 eV in 2NIM with respect to NO_2_. The break of symmetry with respect to the isolated NO_2_ molecule is likely to introduce a chemical shift between the O7/O8 atoms. Theoretically, this has been estimated to be of 0.1 eV. In the experimental XPS spectrum at the O K-edge a small band at about 540.2 eV is clearly visible. To assign this particular feature, accurate MCSCF calculations were performed to include in the theoretical model both the electron relaxation described by the HF method and electron correlation. This was explicitly done for all the outer 12 valence electrons and the energy of the 2NIM ground state, the O(1s) core hole state and the lowest energy shake-up state are reported in the second column of Table 2. The MCSCF calculations predict an energy for the lowest shake-up state (HOMO-LUMO excitation) that is 1.6 eV above that of the core hole state, in excellent agreement with the energy position of the peculiar band observed around 540.2 eV that therefore can be ascribed to a shake-up process. This is a clear example that the HOMO-LUMO energy difference predicted by a calculation for the initial ground state (estimated as large as about 10 eV at the HF level) should be considered only as a very rough approximation for the prediction of the energy level of the lowest shake-up state. In the present case the unusually low energy of the shake-up state derives from the electron distribution of the LUMO orbital in the final state which is mostly located close to the core hole (see next section). Similarly to the 4(5)NIM and related compounds, the O(1s) XPS spectrum shows a broad maximum at about 542 eV. This feature could not be ascribed to the presence of any possible residual gas or fragment from the decomposition of the 2NIM molecule. In the present work we do not have definite assignment for this feature, but in the work of (Feketeová et al., 2015a) it was assigned as a π−π^*^ shake-up.

**Table 2:** Results of MCSCF calculations for: ground state, O core hole state and lowest energy O shake-up state of 2NIM; total energy in second column and relative energy (binding energy) in third column.

**State**	**E(MCSCF)(au)**	**ΔE(eV)**
GS	–428.41814	0.0
O1 corehole	–408.6122987	538.9
O1 shake-up	–408.553992	540.5

### 4.2. The NEXAFS Spectra

**Figure 3:**
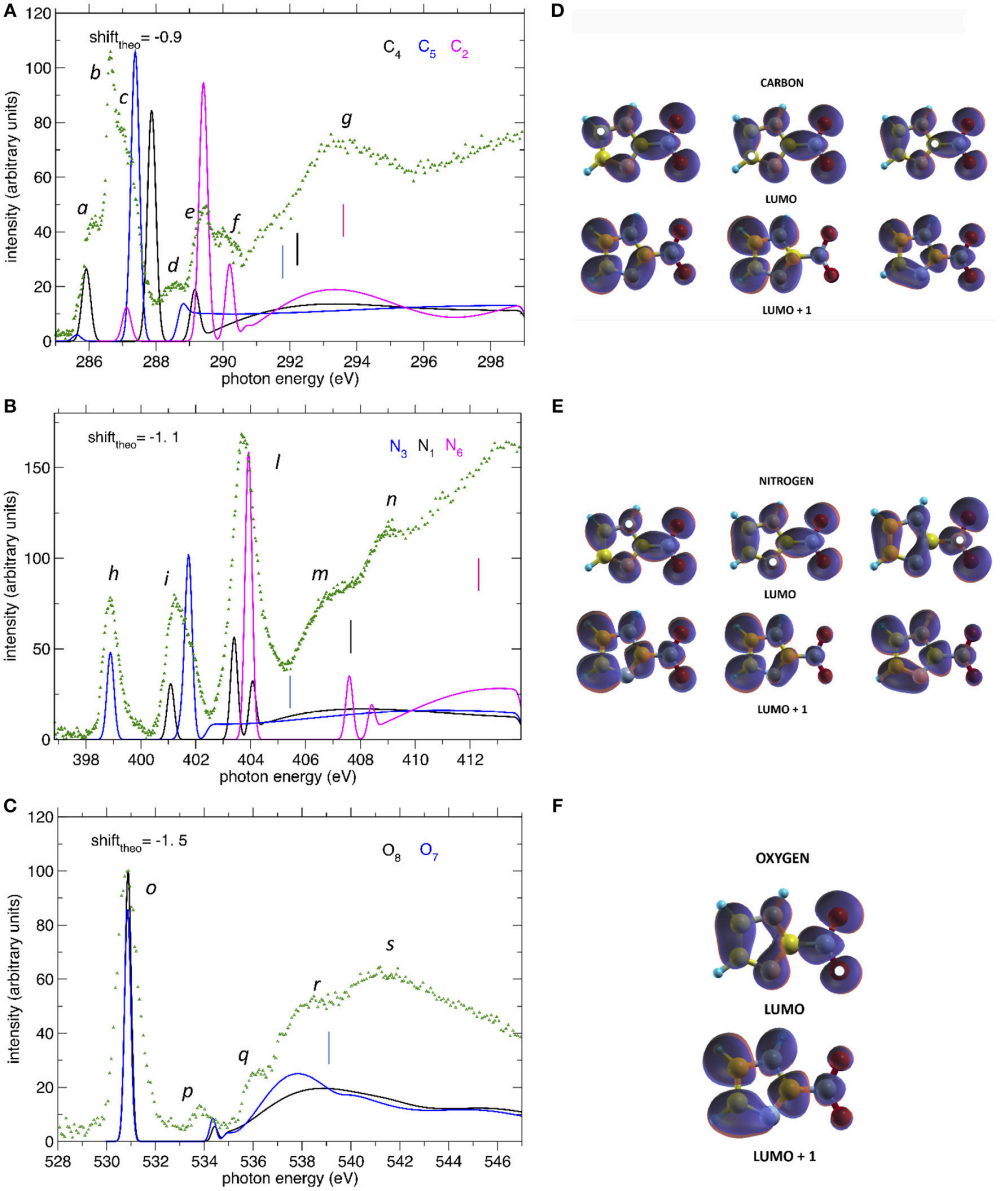
**(A–C)** The NEXAFS spectra of 2NIM measured at the C, N, and O K edge regions (green dots) are compared to the computed ones, displayed with different colors for the contribution of the different non-equivalent atoms of the same species. The shifts applied to the computed spectra and indicated in each figure, produce a good match between experiment and theory for the lowest energy bands. The vertical bars indicate the experimental ionization thresholds from the XPS spectra. **(D–F)** iso-surfaces for the LUMO and LUMO+1 orbitals at all calculated excitation channels. The white dots indicate the sites of excitation.

The comparison between the experimental and theoretical NEXAFS spectra of 2NIM at the C, N, and O K-edges is reported in Figures 3A–C. All the computed spectra were convoluted with a Gaussian function (width = 0.2 eV) to mimic both the experimental response function and the vibrational broadening, and shifted by arbitrary amounts, reported in their respective figures, to obtain a good matching with the low energy bands of the experimental spectra. The calculated spectra neglect the vibrational distribution of the electronic states. This may change the position of the centroid, the shape and the relative intensity of the bands. Nevertheless, there is a very good agreement between theoretical predictions and experimental data in the low energy part of the spectra, i.e., in the region of the excitations involving the LUMO and LUMO+1. Some discrepancies are observed in the region approaching the continuum, due to the poor reproduction of the region of the Rydberg excitation by the adopted theoretical methods. The different colors in Figures 3A–C represent the individual contributions from the non-equivalent atoms and allow identifying the role played by each one in the different energy regions of the spectra, in particular on the discrete features at the low energy side corresponding to excitations to the lowest virtual orbitals. The charge distribution of the LUMO and LUMO+1 orbitals are reported in Figures 3D–F, showing that these orbitals are all of π and antibonding nature. Above the ionization thresholds, broad features usually assigned to transitions to σ^*^ shape resonances are also observed. This kind of excited state involves antibonding σ orbitals quite localized in the core hole region and, as a consequence, the transition dipole moment can be large. However, their usually strong dissociative character, leads to a spreading of such intensity on a large band by a coupling, which depends on the atomic site and the bond environment. In all cases, the excitation energies of the non-equivalent atoms follow the same ordering as in the XPS spectra (see Table 1), even though the charge distribution of the different excited orbitals may produce a different core hole screening for each site and therefore modify the values of the chemical shift with respect to the ones measured in XPS. Concerning the comparison of 2NIM to imidazole, an opposite behavior can be observed in the binding energy shifts of the XPS and NEXAFS spectra. Indeed, while ionization energies of 2NIM shift to larger values with respect to imidazole, the excitation energies shift to a lower one, indicating a stabilization of the LUMO orbitals. This is clearly illustrated in Figure 2, where the binding energies of the LUMO (red bars, assigned according to the theoretical calculation) and of the ionization (black bars) at the C, N, and O K edges are reported for nitrogen dioxide, 2NIM and imidazole. Although in the core excitation the molecule is neutral, the LUMO is embedded in the field of an ion with a localized charge. This leads to a lowering of the LUMO energy with respect to the ground state of the molecule. This stabilization strongly depends on the charge of the core hole and its screening. In the case of 2NIM, the NO_2_ group removes the charge to screen the core hole and the binding energy as well as the attractive force of the partially screened core hole on the LUMO electron increases, thus the LUMO in 2NIM is stabilized with respect to imidazole. This shows that the effect of the substituent group is opposite on the binding energy of the core orbitals and on the excitation energy of the LUMO. Another contribution to the lowering of the LUMO energy in the 2NIM is likely due to the hyperconjugation of the NO_2_ π orbitals with the aromatic orbitals of the imidazole ring. It is not straightforward to evaluate the amount of these two contributions in the observed lowering of the LUMO, but it is realistic to consider that both of them are present.

In the C NEXAFS spectrum (Figure 3A) we observed several structures, labeled *a* to *g*, organized in four main groups. The first group contains the partially resolved features *a* to *c* at 286, 286.6, and 287 eV, respectively. Previously published NEXAFS measurements of 4(5)NIM have empirically assigned this group of peaks (energy region 285.3–287.0 eV), to a series of C(1s) → π^*^ transitions (Feketeová et al., 2015a). In the present work, supported by the theoretical predictions, we assigned the feature *a* mainly to a C5(1s)-LUMO transition with a minor contribution from the C4-LUMO, feature *b* to the C4(1s)-LUMO+1 transition with a minor contribution from the C2(1s)-LUMO and then feature *c* to C5(1s)-LUMO+1 transition. The tiny feature *d* at 288.6 eV is dominated by the C4 contribution while *e* and *f*, at 289.4 and 290.3 eV, respectively, are dominated by core excitations from C2, approaching the C4 and C5(1s) ionization continua. The broad feature *g* at 292–295 eV can be attributed, by present calculations, to σ^*^ resonances, with the strongest contributions due to excitations from the C2 core orbital to antibonding orbitals along C2-N6 and C2-N1 and, to a minor extent, from the C5 core orbital to antibonding orbitals along C5-N1. In the C NEXAFS like in the XPS there was a positive shift, i.e., toward higher excitation energies, of the C2(1s) excitation spectrum with respect to the C4 and C5 contributions, which are very close in energy. The gas phase electron energy loss (EEL) measurement of imidazole (Apen et al., 1993) assigned the overlapping contribution of the LUMO excitations from the three C atoms to a large, unresolved feature at 286.7 eV.

In the N NEXAFS spectrum, three distinct features labeled *h, i*, and *l* at 398.9, 401.1, and 403.9 eV were observed experimentally and were very well reproduced in both position and relative intensities by the theoretical predictions. According to the present calculations, their assignment follows the same ordering as in the XPS spectrum. The first feature, *h*, is attributed entirely to the N3(1s) excitation to the LUMO orbital, the second one, *i*, to the overlapping of the (1s) excitation from N1 to LUMO and from N3 to LUMO+1, while the third and strongest feature, *l*, has a mixed contribution from all three N sites, but is dominated by the N6(1s) to LUMO. The position and assignment of these three features are very close to the ones reported for the 4(5)NIM, i.e., about 400.55, 401.7, and 403.9 eV, respectively (Feketeová et al., 2015a), indicating that the N core excitation is not a sensitive fingerprint to distinguish the nitroimidazole isomers. Similar to the C case, an opposite trend was observed in the core ionization and excitation at the N K-edge. Indeed, the N3 and N1(1s) excitations of imidazole are located at 399.9 and 402.3 eV, respectively (Apen et al., 1993) (i.e., at higher excitation energy with respect to 2NIM) and the N(1s) excitation in NO_2_ is located at an average value of 402.34 eV (Zang et al., 1990; Gejo et al., 2003) (i.e., at lower energy compared to 2NIM). This is the opposite behavior with respect to the XPS, where N3 and N1 (1s) BE were shifted at higher binding energies compared to imidazole, while N6 was shifted toward lower binding energies compared to nitric dioxide. The increased electronic density with respect to NO_2_ results in a decreased binding energy of N6 while, as discussed above, the attraction of the screened hole on the electron of the LUMO destabilizes the LUMO itself. Considering that the hyperconjugation should always have a stabilizing effect on the LUMO, this result indicates that the most relevant effect in determining the stabilization/destabilization of the LUMO is the variation of the hole screening. The *m* and *n* features at around 407 and 409 eV, respectively, can be attributed,using present calculations, to a partial contribution of π^*^ transitions to LUMO+1 and LUMO+2 orbitals from the N6 core orbital as well as to σ^*^ transitions from N1 core orbital to antibonding orbitals along N1-H and N1-C2. Similar broad features were also observed in both imidazole (at 411.4 and 415 eV), where they are attributed to C-N^*^ transitions, and in NO_2_, at 416.16 eV.

At the O K-edge, the NEXAFS spectrum below the ionization continuum is dominated by the intense peak *o* at 530.85 eV, containing the overlapping contribution of the core excitations to the LUMO at atoms O7 and O8. Similarly to the XPS spectrum, the chemical shift between O7 and O8 does not give rise to a measurable shift in the observed peaks, as confirmed by the theoretical prediction of a chemical shift of 0.1 eV (see Table 1). The position of the O(1s) LUMO+1, peak *p* at about 534 eV, is also quite well predicted by the theoretical model. Considering the shift of the O(1s) edge in the theoretical NEXAFS spectra, features *r* and *s*, at around 538 and 541 eV, respectively, can be attributed, by the present calculations, to transitions to σ^*^ orbitals along N6-O.

Concerning the charge distributions reported in Figures 3D–F, the most evident difference between the LUMO and LUMO+1 orbitals can be observed along the C2-N6 bond, which has a bonding/antibonding character in case of excitation to the LUMO/LUMO+1 states, respectively. More subtle differences are present among the different sites of excitation but, since the charge is quite delocalized, no evident correlation between the localization of the core hole and the charge distribution could be made.

### 4.3. Mass Spectra and Partial Ion Yield

**Figure 4:**
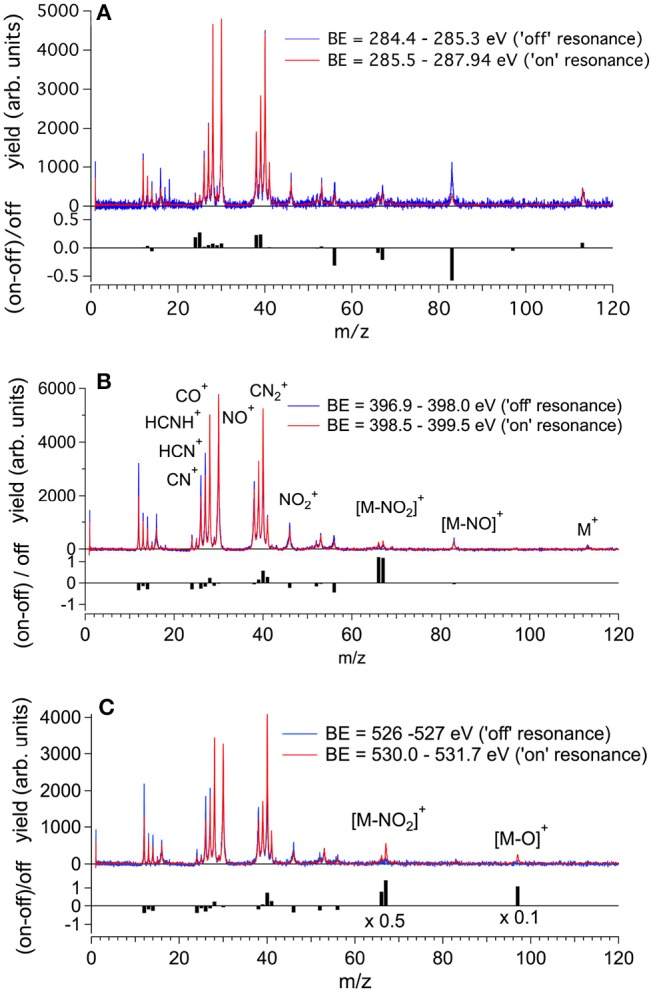
The fragmentation mass spectra of 2NIM measured “on”and “off” resonance at the C, N, and O K-edges (red and blue spectra, respectively) and their weighted difference (gray bars) obtained after a normalization procedure (see text). The binding energy regions 285.5–287.65, 398.5–399.5, and 530.0–531.7 eV have been used to assess the “on” resonance contributions (see Figure 3). The label M indicates the parent ion and the main fragmentation channels of interest in this work are also indicated.

The molecular fragmentation of 2NIM following the C, N, and O core excitation/ionization has been investigated by measuring time-of-flight (TOF) mass spectra at several photon energies across their respective near-edge regions. The mass spectra measured in the energy regions of the transitions from the C, N and O(1s) to the LUMO orbital are reported in Figures 4A–C, respectively, and compared with the ones obtained in the region just below their respective resonance regions. The assignment of the main fragments of interest in the present work is reported in Figure 4 and the list of all fragments is collected in Table S1. In general, the enhanced photoabsorption cross section at the core excitation energies affects the intensity of all fragments, thus the partial ion yields vs. photon energy, mimics the overall shape of the NEXAFS spectrum. However, the fragmentation pattern itself, and therefore the molecular branching ratio, depend on the location of the inner hole (Okada et al., 2003; Tanaka et al., 2006; Céolin et al., 2008; Bernini et al., 2012; Lin et al., 2014, 2015; Salén et al., 2014). To prove this, Figure 4 shows the superposition of the fragmentation mass spectra measured “on” and “off” resonance and the quantity (Yield_*on*_ -Yield_*off*_) / Yield_*off*_ for the major fragments as vertical bars at the bottom of each panel. This quantity allows the variation of the yield “on” resonance, with respect to the “off” resonance one, to be evaluated for each fragment. A value of 1, for example, means that the yield on resonance has doubled, i.e., suffered 100% variation.

Beginning with the 2NIM parent ion (m/z 113) one sees that its contribution to the mass spectra at the three edges is negligible, showing only a minor resonant enhancement in the case of C(1s) → π^*^ excitation (Figure 4A). The fragments at m/z 97 (2NIM-O)^+^, 83 (2NIM-NO)^+^, and 67 (2NIM-NO_2_)^+^ involve the nitro group and share the property of leaving the imidazole moiety unfragmented, even though we cannot infer about its structure as a ring or an open/rearranged feature. Their contribution to the mass spectra is small, however these fragments show significant resonant behavior. At the C(1s) → π^*^ resonance the (2NIM–NO_2_)^+^ displays a noticeable decrease, while the 66^+^ fragment, which corresponds to a further loss of a H atom from (2NIM-NO_2_)^+^, shows a smaller decrease (Figure 4A). The variation of fragment 83^+^ is significant. According to a mass spectrometric and PEPICO study in the valence region (Bolognesi et al., 2016), fragment 83^+^ originates from a (slow) molecular rearrangement of the nitro group, with a swap in position between the O and N atoms, losing the NO group. The reduction of the intensity of this fragment suggests that C(1s) core excitation triggers “faster” fragmentation or decay processes with respect to this “slow” molecular rearrangement, which is therefore hampered. At the N(1s) → π^*^ resonance (Figure 4B), the NO_2_ loss channel does resonate, doubling the intensity of the fragments at m/z 66 and 67, while at the O(1s) → π^*^ resonance (see Figure 4C) both fragments display a very large increase, more than doubling their intensity. At this resonant excitation energy, an even larger increase is observed in fragment m/z 97 due to the O loss. This fragment is barely present in the “off” resonance as well as in the mass spectra at the C and N continua and π^*^ resonances. The complementary fragments ${\text{NO}}_{2}^{+}$ (m/z 46), NO^+^ (m/z 30), and O^+^ (m/z 16) do not display appreciable relative variations in the “on” resonance, a part of the ${\text{NO}}_{2}^{+}$ fragment which suffers a small decrease at the N and O(1s) → π^*^ resonances. This may indicate that (i) the observed NO^+^ and ${\text{NO}}_{2}^{+}$ ions are mostly produced by direct photoionization and/or (ii) the complementary NO and NO_2_ molecules or their fragments are lost as neutral species. These interpretations may be confirmed by a quantitative comparison of the absolute variation of the intensity of the complementary fragments. However, such an estimate based on the present data is unreliable, because it would be affected by the kinetic energy distribution of the involved fragments, which in turn affects the detection efficiency. photoioni-photoion coincidence (PEPIPICO) experiments (Itälä et al., 2017) reported that the dominant contribution in the fragmentation of doubly/multiply charged 4(5)NIM ions just above the C K-edge corresponds to the release of NO^+^, while ${\text{NO}}_{2}^{+}$ is either hardly produced or has a large probability to fragment, consistent with our observations.

**Figure 5:**
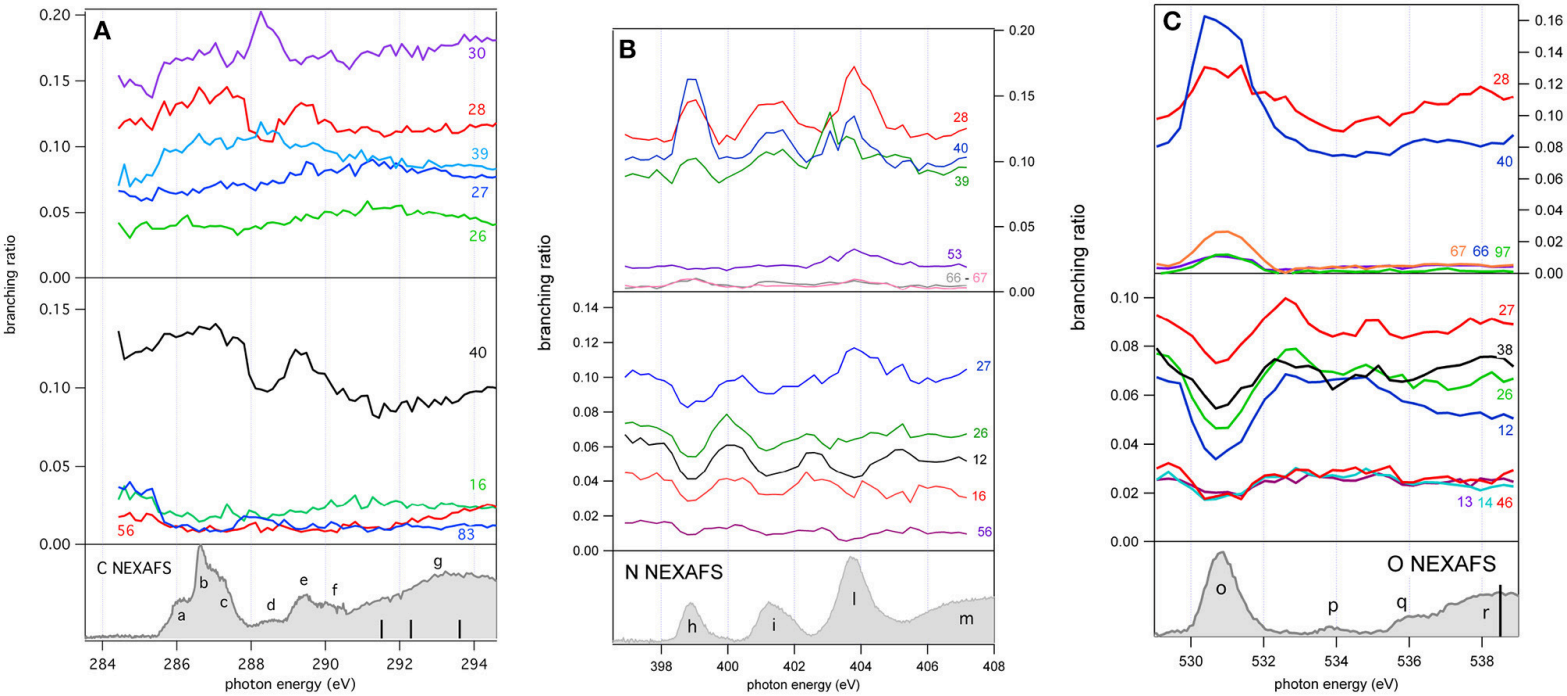
The branching ratios of some selected fragments of 2NIM reported vs. photon energy at the C, N, and O K edge regions. The fragments with a positive or negative variation vs. photon energy are shown in the top and middle panels, respectively. The bottom panels show the corresponding NEXAFS spectra to help identification of the main resonances.

The m/z 56, assigned to the HNCHCO^+^ fragment, is a peculiar fingerprint of 2NIM, not present in 4(5)NIM at least up to 60 eV photon energy (Bolognesi et al., 2016). Similar to 30^+^ and 28^+^, its formation in the VUV energy range is related to the loss of NO and the subsequent fragmentation of the residual 83^+^ fragment. It provides a minor contribution to the mass spectra, with a decrease of intensity at the core excitation, in particular in the C case, Figure 4A. Its branching ratio does not display peculiar resonance effects (see Figures 5A,B). Passing through core excitations and ionizations the fragmentation mechanisms could be different from the ones identified in the valence region, mainly due to the possibility for multiply-charged ion formations. However, the PEPIPICO experiments of (Itälä et al., 2017) in 4(5)NIM at 317 eV photon energy (i.e., still well below the N K edge) do not display any significant signal for ion pairs including m/z fragments heavier than 46. Therefore, we deduced that double ionization events are not the major channels for the production of heavier fragments in 2NIM too.

The largest contribution to the 2NIM mass spectrum was provided by fragments in the m/z regions 38–42 and 24–30. The first region was dominated by the m/z 40 fragment due to C_2_H_2_N^+^ and its correlated species C_2_HN^+^ and C_2_N^+^, while the other region was populated by light species like NO^+^ (m/z 30), CO^+^ /HCNH^+^ (m/z 28) and its correlated species HCN^+^ and CN^+^ due to fragmentation of both the imidazole or the nitro group. All the fragments in the m/z region 38–42 displayed a resonance effect with an increase of their intensity at the three π^*^ excitations, while in the region m/z 24–30 the HCN^+^ and CN^+^ fragments displayed an opposite behavior and decreased their relative intensity at the N and O(1s) → π^*^ resonances, showing how the “localization” of the core hole produces large effects on these fragmentation patterns.

A more complete view of the effect of the excitation of the inner-shell resonances is provided by the branching ratios variation of selected fragments reported vs. photon energy in Figures 5A–C for the C, N, and O K edge regions, respectively, while their values at selected photon energies spanning from 60 (Bolognesi et al., 2016) to 538 eV are reported in Table S1 and displayed in Figure S1.

As already mentioned in the discussion of Figure 4, the 2NIM parent ion (m/z 113) branching ratio decreased significantly with increased photon energy up to the oxygen K edge where its branching ratio vanishes (Figure S1). This trend is very common in molecular species, due to the large amount of energy delivered by inner shell excitation/ionization and to the many active fragmentation channels. Indeed, PEPICO experiments on 2NIM in the VUV energy range, clearly demonstrated that the molecular fragmentation is state-selective; just after the opening of the first fragmentation channel (the NO loss, at around 10.6 eV), the branching ratio of the parent ion drops to zero (Bolognesi et al., 2015, 2016; Cartoni et al., 2018) indicating that the lowest lying molecular orbital is the only one that allows preservation of the parent ion as an intact unit in the photoionization event, at least within a microsecond time scale. Therefore, with increased photon energy the cross section and, as a consequence, the contribution of the electronic ground state to the fragmentation mass spectrum, becomes less and less relevant and eventually negligible. Furthermore, the parent ion does not show any resonant behavior, as discussed above, indicating that the coupling of these core excited states to the cation electronic ground state is very poor. This was confirmed by the photoelectron spectra taken at the three main resonances at the N edge, which showed that the Resonant Auger process does not lead to an appreciable population of the cation HOMO state. Among the three regions shown in Figure 5, the carbon region (Figure 5A) is the one that displays the smallest variations. Weak resonance effects are observed in the region of the *a-c* resonances (see Figure 3A for the labeling), while some effects on m/z 30, 28, and 40 are observed in correspondence of the *d-f* resonances, with an enhancement of the m/z 30 branching ratio and a depletion on the other two.

**Figure 6:**
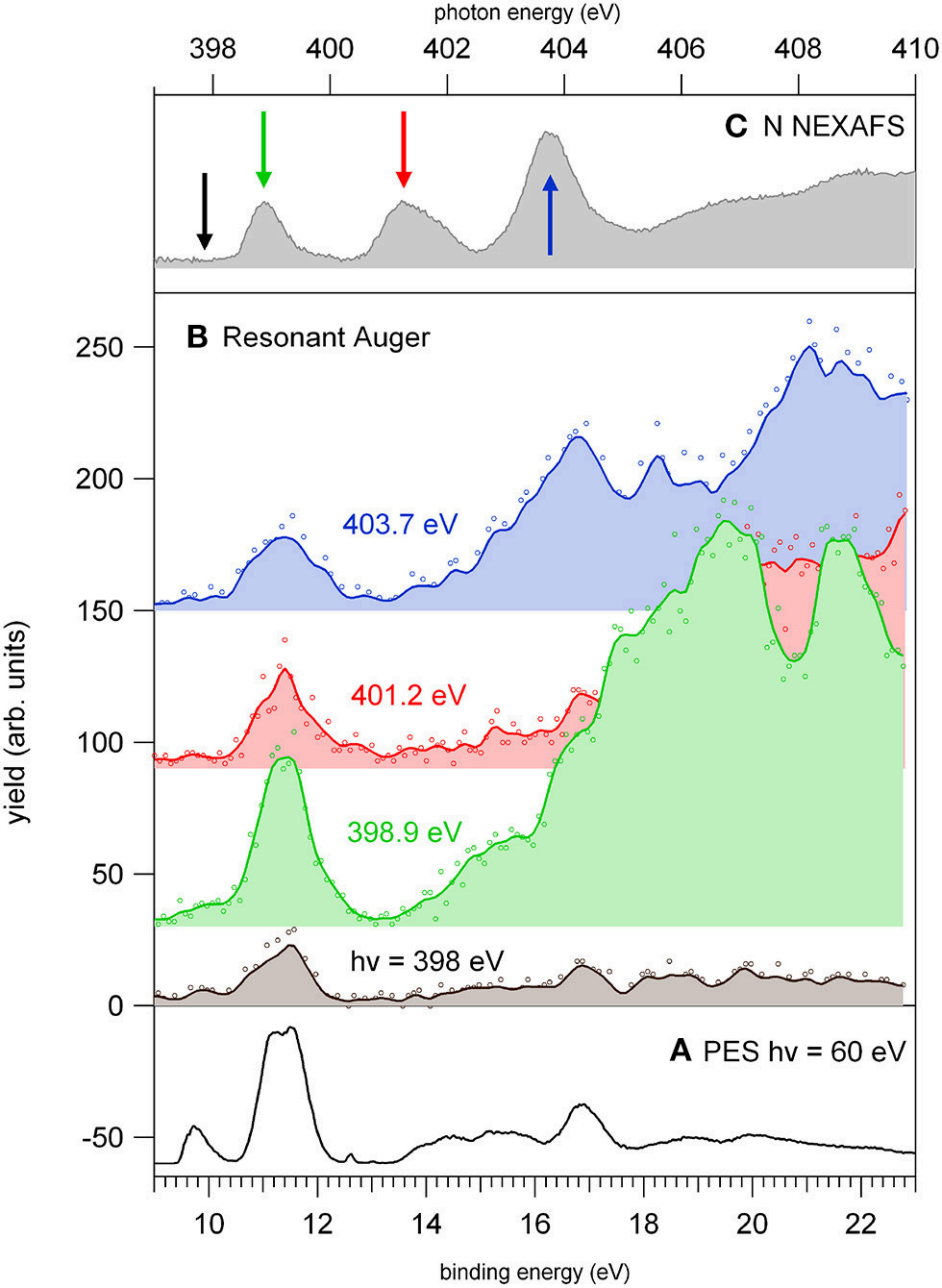
The photoelectron spectrum of 2NIM measured **(A)** at 60 eV photon energy and **(B)** at four photon energies around the N K-edge, respectively, in the continuum below the LUMO (black) and at the three major features of the NEXAFS spectrum **(C)**. The photon energies of the three spectra are green, red, and blue for increasing photon energy, and their corresponding feature in the NEXAFS spectrum is indicated in figure panel **(C)**. The continuum lines represent 5-point smooth of the data.

In this region, according to the calculations, the main process is due to the population of the LUMO involved in the excitation of the C2 atom, the one directly bound to NO_2_. In the region of the nitrogen inner shell resonances (Figure 5B), the branching ratios of most of the fragments display resonance effects, attenuated in the case of m/z 53, 66, and 67. It is interesting to observe that at the *h* resonance, attributed to the population of the LUMO by the promotion of an electron from the N3(1s) orbital, the effects are opposite for the m/z 28, 39, and 40 and the m/z 12, 16, 26, 27, and 56. This opposite behavior is also observed in the region of the *i* resonance, but seems to disappear at the *l* resonance, where mainly the excitation of the LUMO with the core hole in the N6 site occurs. The observations in the oxygen region (Figure 5C) are similar, with large effects effects in the region of the *o* resonance. The branching ratios of fragments m/z 40 and 28 display a clear decrease for increasing photon energy (Figure S1) with an interesting enhancement on-resonance (Figure 5). These observations may suggest the important role of valence/inner valence fragmentation processes in the production of these fragments. The cross section of valence/inner valence states decreases at larger photon energies and in the core ionization continua, but shows significant enhancements at the core resonant excitations, where the valence shell states can be efficiently populated via Resonant Auger emission as shown in the N case, for example (Figure 6).

The resonant photoemission leads to a preferential population of the inner valence states (via spectator Resonant Auger decay) as compared to the direct photoemission measured just below the first resonance. In the binding energy region 13–18 eV, the PEPICO experiments (Bolognesi et al., 2016; Cartoni et al., 2018) have shown that the fragmentation is dominated by the release of fragments 28^+^, 40^+^. In the valence single ion states, fragment 40^+^ originates by a chain of events where, following the NO_2_ loss, the subsequent fragmentation of fragment 67^+^ by HCN loss, leads to the HNCCH^+^ fragment, that can exist both in its linear or ring structure (Bolognesi et al., 2016; Cartoni et al., 2018). Formation of fragment 28^+^, instead, is triggered by the NO loss, leading to fragment 83^+^ that, after several molecular rearrangements and the release of HCN and CO neutral species, ends up in the HCNH^+^ fragment at m/z 28. Moreover, at these core excitation energies, additional mechanisms of formation involving the fragmentation double and multiply charged ions, may also become active. A general trend is that for increasing photon energy the branching ratios of “small” fragments (m/z ≤ 30, with the exception of m/z 28) increases in comparison to the larger ones (see m/z 12 and 30 in Figure S1). Their branching ratios increase as new core ionization thresholds are crossed, while they decrease at resonant excitation energies. Considering the many pathways that could produce these fragments, their detailed discussion would be uncertain. However, they likely result from a stepwise fragmentation process, involving highly excited singly or multiply-charge ions. Thus, the observed difference can be explained by the formation of excited dications via Auger decay when the excitation occurs above the K-edge, while on resonance, the Resonant Auger Electron processes populate cation states, hampering fragmentation patterns that require larger amount of energy.

## 5. Conclusions

The core excitation and ionization of 2NIM has been investigated by a set of complementary experimental techniques, which span from electron spectroscopies to mass spectrometry and by accurate computational methods for the prediction of photoemission and photoabsorption spectra. The XPS spectra can be interpreted at a qualitative level, by a building block approach with imidazole and nitrogen dioxide as constituents of nitroimidazoles; this assignment has been confirmed by theoretical simulations of the spectra. The electronegativity of the NO_2_ ligand withdraws charge from the imidazole ring affecting its stability. We observed, in fact, that an initial state picture, corresponding to the KT approximation, may provide a rough, sometimes misleading, prediction of the chemical shifts in NIM. A reasonable prediction can only be obtained considering final state effects (polarization and screening due to the increased/decreased number of electrons around the ionization site). Moreover, the building block approach must be considered with caution when one of the constituents of the larger molecule, such as the NO_2_ ligand in 2NIM, may change geometry (${\text{NO}}_{2}^{+}$ is straight in gas phase and bent in 2NIM) and electronic structure (NO_2_ is a radical, while 2NIM is not). While the core levels can be qualitatively related to the two different components, chemically shifted from the imidazole and NO_2_ value by the predictable effect of the nitro group electronegativity, only ΔDFT calculations could confirm the qualitative assignment and the relative values of the chemical shifts. This can be related to the recent observation in the photofragmentation spectra of 2NIM where, at least in the VUV energy region, the NO-loss was the most favorable fragmentation channel, from which all others followed. An intense “shoulder” observed on the high energy side of the main peak in the O(1s) photoemission spectrum, was assigned, by accurate MCSCF calculations, to the lowest shake-up state (HOMO-LUMO excitation) that is predicted to be located at an energy 1.6 eV above that of the core hole state. The unusually low energy of the shake-up state derives from the electron distribution of the LUMO orbital in the final state, which is mostly located close to the core hole.

In the NEXAFS spectra, the combined experimental and theoretical study provided the observation and assignment of the major features due to core electron excitation from the different, and well identifiable, atomic sites in the molecule as well as a description of the corresponding charge distribution in the LUMO and LUMO +1 orbitals. A stabilization of the LUMO has been observed in 2NIM with respect to imidazole. In the mass spectrometry experiments, the tunability of the photon energy has been used to follow the evolution of the partial ion yields across the different core excited/ionized states of the molecule. Significant effects, especially for channels involving the release of the nitro group, were observed in terms of a variation of the branching ratios in the investigated regions. The cases of the NO_2_, NO, and O losses provide clear evidence of a correlation between the localization of the vacancy and the fragmentation mechanism. This may be considered as possible evidence of a “*molecular knife”* picture. On the other hand, for the smaller fragments, the observed effects could be rationalized considering that the preferential decay of core excited states is the Resonant Auger decay, which populates the cation states in the valence/inner valence region. The leading mechanism is therefore more of a “*memory effect,”* ruled by the coupling of the inner shell electronically excited state to the valence/inner valence states, and their following fragmentation.

## Data Availability

All datasets generated for this study are included in the manuscript and/or the Supplementary Files.

## Author Contributions

PB, PM, ST, BM, RR, and LA performed the experiment. VC, LS, GB, and SM performed the theoretical calculations. AC, MC, and JC participated in the data analysis and interpretation. PB and LA planned the experiment and prepared the manuscript. All authors contributed to the interpretation of the results and the revision of the manuscript.

### Conflict of Interest Statement

The authors declare that the research was conducted in the absence of any commercial or financial relationships that could be construed as a potential conflict of interest. The reviewer LE declared a past co-authorship with one of the authors VC to the handling editor.
