# Supplementary material for: Core Shell Investigation of 2-nitroimidazole
Source: Front Chem. 2019 Apr 2;7:151. doi: 10.3389/fchem.2019.00151 (PMC6454003; doi:10.3389/fchem.2019.00151)
Supplement: Supplementary file 1 [file Data_Sheet_1.PDF]

Supplementary Material

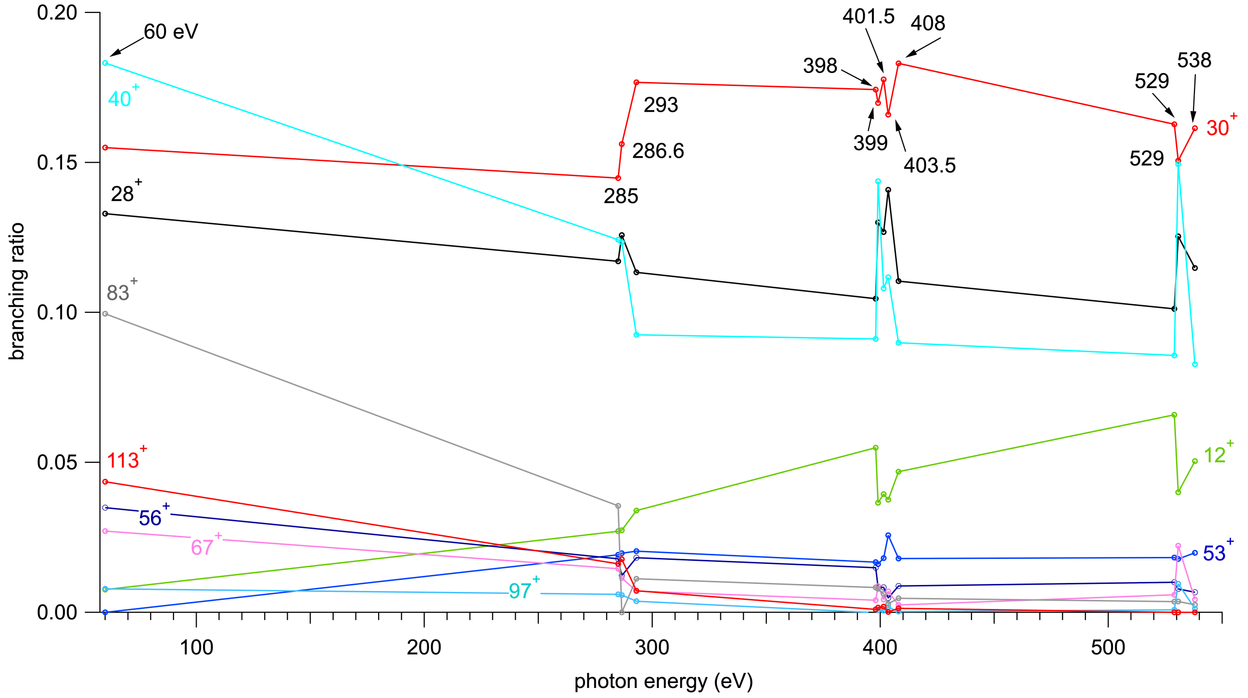

Figure S1: Overview of variation branching ratio of some selected fragments at selected photon energies from Table SI.1 reported between 60 and 538 eV, across C, N and O continua and resonant excitations. The data at 60 eV (Bolognesi et al., 2016) have been measured with the same experimental apparatus and conditions of the present measurement.

Table S1. The branching ratio of the main 2NIM fragments (> 0.1) at some selected photon energy (in the conitnuum and on resonance) between the 60 eV (Figure 1 of (Bolognesi et al., 2016)) and 539 eV. The proposed assignment is just an indication and, whenever possible, it does relay on calculations performed in the VUV photon energy range.

		Photon Energy (eV)											
**m/z**	Assignment	60	285	286.6	293	398	399	401.5	403.5	408	526	529	538
**12**	C^+	0.76	2.71	2.74	3.39	5.49	3.65	3.94	3.75	4.69	6.59	4.00	5.04
**13**	CH^+	0.87	1.75	1.81	2.01	2.32	1.99	2.28	1.95	2.67	2.56	2.07	2.53
**14**	CH_2^+/N^+	0.53	1.34	1.26	1.30	2.30	1.63	1.98	1.28	1.97	2.63	1.93	2.25
**24**	C_2^+	0.07	0.53	0.63	0.61	0.69	0.49	0.64	0.42	0.88	1.10	0.68	0.91
**25**	C_2H^+	0.25	0.73	0.93	0.89	0.65	0.66	0.88	0.76	1.14	0.93	0.81	1.00
**26**	$C_2H_2^+/CN^+$	1.25	3.82	3.90	4.81	6.48	4.81	5.41	5.42	6.14	7.42	5.14	6.94
**27**	HCN^+	3.20	6.18	6.48	8.09	9.16	7.64	8.24	9.87	9.06	9.01	7.80	9.13
**28**	$CO^+/HCNH^+$	13.29	11.71	12.58	11.33	10.46	13.00	12.68	14.09	11.04	10.12	12.53	11.48
**29**	H_2CNH^+	1.22	1.28	1.31	1.30	1.43	1.25	1.25	1.12	1.24	1.18	1.17	1.09
**30**	NO^+	15.49	14.48	15.61	17.67	17.43	16.99	17.77	16.59	18.30	16.27	15.06	16.14
**38**		2.70	5.65	6.93	7.45	6.96	6.57	7.24	7.99	7.87	7.32	5.95	7.57
**39**		6.31	7.63	9.44	8.63	7.87	9.07	9.51	10.28	8.51	7.44	8.02	8.48
**40**	$C_2H_2N^+$	18.32	12.42	12.34	9.26	9.11	14.37	10.80	11.17	8.99	8.56	14.94	8.27
**41**		3.14	3.17	3.21	2.94	2.72	3.50	2.93	2.66	2.37	2.67	3.45	2.39
**46**	NO_2^+	2.38	2.85	2.86	4.04	3.35	2.61	2.65	1.80	3.80	3.08	1.98	2.40
**52**		2.31	1.00	0.99	1.10	0.83	0.70	0.74	0.99	0.79	1.07	0.80	0.99
**53**		–	1.92	1.97	2.04	1.67	1.61	1.81	2.57	1.79	1.82	1.78	1.98
**56**	$HNC(H)CO^+$	3.49	1.78	1.22	1.82	1.49	0.84	0.83	0.48	0.88	1.00	0.78	0.67
**66**	$(2NIM\text{-}H\text{-}NO_2)^+$	1.06	1.14	1.04	0.72	0.43	0.95	0.63	0.64	0.36	0.37	0.96	0.41
**67**	$(2NIM\text{-}NO_2)^+$	2.71	1.45	1.14	0.71	0.40	0.87	0.43	0.70	0.24	0.58	2.22	0.43
**83**	$(2NIM\text{-}NO)^+$	9.96	3.55	–	1.12	0.83	0.79	0.62	0.28	0.47	0.36	0.36	0.25
**97**	$(2NIM\text{-}O)^+$	0.78	0.60	0.57	0.37	0.01	0.07	0.06	0.17	0.03	0.08	0.95	0.12
**113**	$2NIM^+$	4.36	1.62	1.77	0.72	0.10	0.16	0.19	0.01	0.13	–	–	–

REFERENCES

Bolognesi, P., Casavola, A., Cartoni, A., Richter, R., Markus, P., Borocci, S., et al. (2016). Communication: Position does matter: The photofragmentation of the nitroimidazole isomers. *J. Chem. Phys.* 145, 191102
